# Evaluation of Antimicrobial and Wound Healing Potential of *Justicia flava* and *Lannea welwitschii*

**DOI:** 10.1155/2013/632927

**Published:** 2013-09-15

**Authors:** Christian Agyare, Solomon Boamah Bempah, Yaw Duah Boakye, Patrick George Ayande, Martin Adarkwa-Yiadom, Kwesi Boadu Mensah

**Affiliations:** ^1^Department of Pharmaceutics, Faculty of Pharmacy and Pharmaceutical Sciences, Kwame Nkrumah University of Science and Technology, Kumasi, Ghana; ^2^Center for Discovery and Innovation in Parasitic Diseases, Department of Pathology, University of California San Francisco, San Francisco, CA 94158-2330, USA; ^3^Department of Human Biology and Nursing, School of Biological Sciences, University of Cape Coast, Cape Coast, Ghana; ^4^Drugs and Forensic Laboratory, Ghana Standards Authority, Accra, Ghana; ^5^Department of Pharmacology, Faculty of Pharmacy and Pharmaceutical Sciences, Kwame Nkrumah University of Science and Technology, Kumasi, Ghana

## Abstract

Microbial infections of various types of wounds are a challenge to the treatment of wounds and wound healing. The aim of the study is to determine the antimicrobial, antioxidant, and *in vivo* wound healing properties of methanol leaf extracts of *Justicia flava* and *Lannea welwitschii*. The antimicrobial activity was investigated using agar well diffusion and microdilution methods. The free radical scavenging activity of the methanol leaf extracts was performed using 1,1-diphenyl-2-picryl-hydrazyl (DPPH). The rate of wound contraction was determined using excision model. The test organisms used were *Escherichia coli* ATCC 25922, *Pseudomonas aeruginosa* ATCC 4853, *Bacillus subtilis* NTCC 10073, *Staphylococcus aureus* ATCC 25923, and clinical strains of *Candida albicans*. The MICs of methanol leaf extract of *J. flava* against test organisms were *E. coli* (7.5 mg/mL); *P. aeruginosa* (7.5 mg/mL); *S. aureus* (5 mg/mL); *B. subtilis* (7.5 mg/mL); and *C. albicans* (5 mg/mL). The MICs of methanol leaf extract of *L. welwitschii* against test organisms were *E. coli* (5 mg/mL); *P. aeruginosa* (10 mg/mL); *S. aureus* (5 mg/mL); *B. subtilis* (2.5 mg/mL); and *C. albicans* (2.5 mg/mL). The MBC/MFC of the extract was between 10 and 50 mg/mL. The IC_50_ of the reference antioxidant, **α**-tocopherol, was 1.5 **μ**g/mL and the methanol leaf extracts of *J. flava* and *L. welwitschii* had IC_50_ of 65.3 **μ**g/mL and 81.8 **μ**g/mL, respectively. The methanol leaf extracts of *J. flava* and *L. welwitschii* gave a significant reduction in wound size as compared to the untreated. The rates of wound closure after the application of the extracts (7.5% w/w) were compared to the untreated wounds. On the 9th day, *J. flava* extract had a percentage wound closure of 99% (*P* < 0.01) and that of *L. welwitschii* exhibited wound closure of 95% (*P* < 0.05) on the 13th day compared to the untreated wounds. The two extracts significantly (*P* < 0.01) increased the tensile strength of wounds compared to the untreated wounds. The extracts treated wound tissues showed improved angiogenesis, collagenation, and reepithelialization compared to the untreated wound tissues. The preliminary phytochemical screening of *J. flava* and *L. welwitschii* leaf extracts revealed the presence of tannins, alkaloids, flavonoids, and glycosides. The above results indicate that methanol leaf extracts of *J. flava* and *L. welwitschii* possess antimicrobial and wound healing properties which may justify the traditional uses of *J. flava* and *L. welwitschii* in the treatment of wounds and infections.

## 1. Introduction

Traditional medicinal practices are important part of the primary healthcare delivery system in most of the developing countries. According to the World Health Organization, an estimated 3.5 billion people from developing countries in Africa and Asia depend on plants as part of their primary healthcare. Many African people depend on traditional medicine for their health problems, including various forms of wounds and microbial infections [1, 2]. Many rural and some urban people in West Africa countries including Nigeria, Benin, Ghana, and Niger still depend on herbs for their health needs [3]. Due to the high cost of effective drugs from hospitals and pharmacies as well as very low levels of availability of curative drugs, useful medicinal plants are sold in markets in most African countries. 

Wounds are a major cause of physical disabilities. Wound healing process consists of different phases which are coordinated in such way to restore the integrity of the damaged tissue. Wound healing can be adversely affected by many factors such as oxidants, inflammation, and microbial infections. Wound is a break in the skin layer or covering and the wound healing is a complex and dynamic process of restoring cellular structures and tissue layers. The process of wound healing is a response to the injury that sets into motions a sequence of events, and there are basically four phases of the healing process, namely, haemostasis, inflammatory, proliferative, and remodeling phases which depended on each other [4]. Different kinds of wounds may be treated differently from one another, depending upon how they occur and the extent of the injury.

Plants and their extracts have been used for the treatment of skin disorders including wounds for centuries [5]. The presence of these microorganisms affects the healing process and can lead to the formation of chronic wounds. Staphylococcal and streptococcal wound infections are common because they are nearly always present on the skin, and these can lead to the infection of wounds [6]. Because of increasing antimicrobial resistance and infection of wounds by pathogenic organisms, plant extracts are of new interest as antiseptics and antimicrobial agents [4]. 

*Lannea welwitschii* (Hiern) Engl. which belongs to the family Anacardiaceae is found growing in deciduous and secondary forests of Africa from Côte d'Ivoire to Cameroons and extending to Uganda and Angola. And it is locally known in Asante-Twi language in Ghana as “Kuntunkuri.” Decoction of the leaves is used for the treatment of diarrhea, dysentery, swellings, gout, gingivitis, topical infections, and wounds [1]. The roots are used for food poisoning, nasopharyngeal infections and as emetics. The stem bark has been found to contain glycosides, tannins, and saponins [7, 8]. The bark has been found to possess antidiarrhoeal property [9] and two cytotoxic compounds; namely, lanneaquinol and 2′(R)-hydroxylanneaquinol have been isolated from the plant [10].

*Justicia flava *(Forssk.) Vahl of the family Acanthaceae is found growing in disturbed habitat, on a wide range of soil types and in full sun or semishady areas. It is widespread in tropical and southern Africa. It is called “Afema” in local Asante-Twi language in Ghana. It is used in traditional medicine for the treatment of cough, paralysis, fever, epilepsy, convulsion and spasm, and skin infections and disorders. The roots are also used for diarrhea and dysentery [1, 11]. For recent times, no pharmacological activity has been reported for *J. flava* despite its popular uses in traditional medicine [12]. Steroids including campesterol, stigmasterol, sitosterol, and sitosterol-D-glucoside were isolated from the leaves and roots of *J. flava *[13–15]. The study was designed to perform preliminary phytochemical screening and investigate the antimicrobial, antioxidant, and wound healing activities of the methanol leaf extracts of *L. welwitschii* and *J. flava *based on the ethnobotanical uses of these plants.

## 2. Materials and Methods

### 2.1. Plant Materials and Chemicals

Leaves of *J. flava* and *L. welwitschii* were collected in May 2011 from Krofrom in the Atwima-Kwanwoma district of Ashanti Region and authenticated by Dr. A. Asase of Ghana Herbarium, Department of Botany, University of Ghana. Voucher specimens of the plants have been deposited at Ghana Herbarium, University of Ghana, Ghana. The various plant materials were dried at room temperature (28–30°C) for two weeks. The dried parts were then powdered. Unless stated otherwise, all the chemicals and reagents were bought from Sigma (Deisenhofen, Germany).

### 2.2. Preparation of Extracts

The fresh leaves of *J. flava* and *L. welwitschii* were thoroughly washed with tap water to remove dirt and soil particles. The plant materials were air-dried between 30 and 38°C for 5 days and then powdered using laboratory mill machine (Type 8, Christy and Norris Limited, UK). Twenty-five grams of the powdered leaves of *J. flava* were added to 300 mL of 70% v/v methanol and extracted with Ultra-Turrax T 50 (Janke & Kunkel, Labortechnik, Germany) under ice-cooling at a speed of 24000 rpm for 3–5 min. The mixture was filtered using Whatman filter paper no. 10. The rotary evaporator was then used to concentrate the supernatant below 40°C and lyophilized. The above extraction procedure was repeated for powdered leaves of *L. welwitschii*. The yields of the methanol leaf extract of *J. flava* (JFL) and methanol leaf extract *L*. *welwitschii* (LWL) were 11.6 and 9.8% w/w (related to the dried material), respectively.

### 2.3. Preliminary Phytochemical Screening

Preliminary phytochemical screening on methanol leaf extracts of *J. flava *and* L. welwitschii *was done to determine the presence of starch, tannins, glycosides (sapogenetic, anthracene, and cyanogenetic), flavonoids, steroids, and alkaloids [16–18]. The total amount of tannins present in the dried powdered leaf materials was determined according to the method described by Glasl (1983) [19] using pyrogallol (Merck, Darmstadt, Germany, purity 99.5%, HPLC) as reference compound.

### 2.4. HPLC Finger-Printing of Extracts

The HPLC finger-printing of JFL and LWL was determined with Thermo Finnigan HPLC system using Hypersil Gold C_18_, reversed-phase column (150 × 4.6 mm). The concentration of extracts used was 10 mg/mL. HPLC optimum conditions were injection volume: 10 *μ*L, detection wavelength: 254 nm, mobile phase: 0.1% acetic acid : acetonitrile/60 : 40 v/v (isocratic condition), temperature: 22°C, pump pressure: 28 MPa, flow rate: 1 mL/min, and running time: 10 min.

### 2.5. Determination of Antimicrobial Activity of Extracts

#### 2.5.1. Agar Diffusion Method

The antimicrobial activities of the methanol leaf extracts (JFL and LWL) and reference drugs (chloramphenicol and clotrimazole) were determined according to the method described by Agyare et al. [18]. Sabouraud agar and nutrient agar (Oxoid Limited, UK) media were used to determine the antifungal and antibacterial activities of the extracts, respectively. One hundred microliter (10^6^ cfu/mL) of the test organisms (*Staphylococcus aureus* ATCC 25923, *Bacillus subtilis *NCTC 10073, *Escherichia coli *ATCC 25922, *Pseudomonas aeruginosa *ATCC 27853, and a clinical strain of *Candida albicans*) were used to seed 20 mL nutrient agar and sabouraud agar plates, respectively. In each of these plates, four (4) equidistant wells with diameter of 8 mm were made, the wells were filled with different concentrations of the extracts, and reference drugs were dissolved in dimethyl sulfoxide (DMSO) and allowed to diffuse at room temperature (28–30°C) for 1 h. The zones of growth inhibition were measured after 24 h incubation at 37°C (for bacterial strains) and 3 days at 30°C (for the fungus *C. albicans*). The solvent (DMSO) alone was used as negative control and it exhibited no antimicrobial activity against the test organisms. The minimum bactericidal concentration (MBC) and minimum fungicidal concentration (MFC) were determined as the minimum concentration of extracts where no growth occurred when a small part of seeded agar without growth was streaked on 20 mL nutrient agar and sabouraud agar in a Petri dish for bacterial test organisms and *C. albicans*, respectively, and then incubated at 37°C for 24 h.

#### 2.5.2. Microdilution Method

 The MIC of JFL and LWL against the test bacteria were determined according to the modified microdilution method described by Agyare et al. [18] and Eloff [20]. Extracts (100 mg/mL) were prepared using DMSO and test solutions (25–100 *μ*L) were serially diluted to 100 *μ*g/mL and then transferred into the 96 wells of the microtitre plates. One hundred microliter of 10^6^ cfu/mL of the test bacteria grown in nutrient broth was added to each well in the microplates. The plates were covered and then incubated at 37°C for 24 h. Thirty microlitre of 125 mg/mL of 3-(4,5-dimethylthiazol-2-yl)-2,5-diphenyltetrazolium bromide (MTT) was added to each well and then incubated at 37°C for 30 min in order to detect the growth or survival of organisms or otherwise. *C. albicans* was cultivated in sabouraud dextrose broth (Oxoid Limited, UK) and then incubated for 3 days at 30°C. The MICs of JFL and LWL extracts against the test fungus were determined according to the guidelines prescribed in the National Committee for Clinical Laboratory Standards [21] for filamentous fungi. The MICs of the extracts against *C. albicans* were detected as the minimum concentration of extracts that exhibited the microbial growth after the addition of MTT to the medium and plates incubated at 37°C for 20 min [22]. The experiments were repeated three times. 

### 2.6. Determination of Free Radical Scavenging Activity

Antioxidant activities of the extracts were determined by the method described by Chizzola et al. [23] using 1,1-diphenyl-2-picryl-hydrazyl (DPPH). Hundred micromolar (0.1 mM) of DPPH in methanol was prepared and 10 *μ*L was added to each 100 *μ*L of methanol extracts (JFL and LWL) with *α*-tocopherol as reference antioxidant at different concentrations in 96-well microtitre plates. The plates were shaken for 30 s and their absorbance was determined at 517 nm after 30 min. The inhibition percentage (%) of radical scavenging was determined using the following equation: Inhibition (%) = [(*A*_0_ − *A*_1_)/*A*_0_] × 100, where *A*_0_ is the absorbance of the control, *A*_1_ is the absorbance of the sample at 517 nm, and inhibitory concentration, IC_50_, is the amount (*μ*g/mL) reducing the absorbance by 50%.

### 2.7. Evaluation of Wound Healing Properties

#### 2.7.1. Experimental Animals

Twenty-five male and female Sprague-Dawley rats were kept in stainless steel cages and served with normal commercial rats diet (GAFCO Ltd., Tema, Ghana), given water *ad libitum* and maintained under laboratory conditions (temperature 28–30°C, relative humidity 60–70%, and normal light-dark cycle). The rats were accustomed in the laboratory two days before the experiment to experimenter handling and the apparatus to reduce the effects of stress. All techniques and methods used in this study were performed in accordance with the National Institute of Health Guidelines for the Care and Use of Laboratory Animals (NIH, Department of Health Services Publication no. 83-23, revised 1985). The protocols for the study were approved by the Department of Pharmacology Ethics Committee, Faculty of Pharmacy and Pharmaceutical Sciences, Kwame Nkrumah University of Science and Technology, Kumasi, Ghana.

#### 2.7.2. Excision Wound Model

The twenty-five male and female Sprague-Dawley rats weighing 115–120 g were anaesthetized with ketamine at a dose of 120 mg/kg body weight subcutaneously before the wounds were created. The dorsal furs of the animals were shaved to a circular diameter of about 40 mm with the aid of razor blades, and the anticipated area of the wound was marked on the shaved skin. The areas were cleaned with 70% ethanol before the excision wounds were created [24]. Skin wounds were created with the aid of toothed forceps, surgical blades, and pointed scissors. The wounds were left opened and the animals divided into five (5) groups of five animals each. The first group was topically treated with 1% w/w silver sulphadiazine cream (Ayrton Drugs, Ghana) as reference drug [25]. The second group was treated with aqueous cream (vehicle alone). The third group was left untreated and allowed for normal wound healing process. The last two groups were treated with 7.5% w/w extract aqueous creams JFL and LWL, respectively. The aqueous cream of the extracts (7.5% w/w of JFL and LWL) were freshly prepared daily and used for the treatment of the wounds. The 7.5% w/w extract aqueous creams of the extracts was selected and used for the wound healing studies because this concentration of the extracts was the lowest concentration that exhibited highest antimicrobial activity using the agar well diffusion method [18] as described above (data not shown). Wound treatment commenced on the second day after wound creation. The extracts and reference drugs were topically applied to the wounds 24 hourly for 24 days. In the course of treatment, scaled photographs of the wound areas were taken (by means of high-resolution Olympus digital camera, Cameron Sino, Hong Kong) alongside a millimeter scale measurement every 48 h starting from the first day of wound treatment. The wound areas were determined every two days till the 24th day.

Another set of twenty-five male and female Sprague-Dawley rats weighing 115–120 g were grouped into five groups of five animals each, then anaesthetized with ketamine at a dose of 120 mg/kg body weight, treated as described above for the excision wound model, and then used for the determination of tensile strength and histological examinations of the wound tissues.

#### 2.7.3. Measurement of Tensile Strength

Tensile strength shows how much the restored tissues resist breaking under tension and may show the strength of the healed tissue. The tensile strength was measured according the method described by Shivhare et al. [25] and Kuwano et al. [26] at the 10th day after wounding. The newly formed tissue including scar was excised and the tensile strength was measured with the help of tensiometer (Krüss GmbH, Germany). The wound breaking strength was measured as the weight of water at the time of wound breaking per area of the specimen.

### 2.8. Histopathological Examinations

Wound tissue specimens from untreated and treated rats were taken at day 14 (after treatment). The tissue specimens were taken on 14th day (after treatment) so that the influence of the extracts and reference drugs on skin cells and its components can be evaluated well instead of last day of complete wound closure where there will not much difference in the skin tissues and structures. The cross-sectional full-thickness wound scar of about 5 mm thick sections from each group were collected for the histopathological evaluation [27]. Samples were fixed in 10% buffered formalin for 24 hours and dehydrated with a solution of sequence of ethanol-xylene series, processed and then blocked with paraffin at 40–60°C, and sectioned into 5-6 *μ*m thick sections. The sections were stained with hematoxylin and eosin stain. Collagen deposition was identified by staining with the sections with Van Gieson's stain. Mast cells were stained with toluidine blue [28].

### 2.9. Statistical Analysis

GraphPad Prism Version 5.0 for Windows (GraphPad Software, San Diego, CA, USA) was used for all statistical analyses. Data are presented as mean ± SEM (*N* = 5) and analyzed by one-way ANOVA followed by Dunnett's multiple comparison test. *P < 0.05, **P < 0.01, and ***P < 0.001 were considered statistically significant in all analyses. The graphs were plotted using Sigma Plot for Windows Version 11.0 (Systat Software Inc., Germany).

## 3. Results

### 3.1. Preliminary Phytochemical Screening

Methanol leaf extracts from *J. flava* (JFL) and *L*. *welwitschii* (LWL) were found to contain tannins (with varying amounts), steroids, flavonoids, alkaloids, sapogenetic glycosides, and carbohydrates (Table 1). 

### 3.2. HPLC Finger-Printing of Extracts

**Figure 1:**
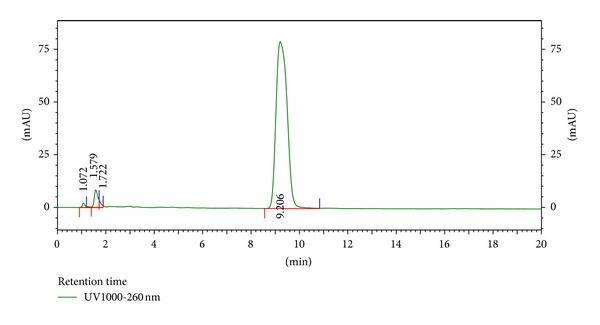
HPLC chromatogram (finger-printing) of methanol stem bark extract (JFL) of *J. flava *at *λ* = 254 nm.

**Figure 2:**
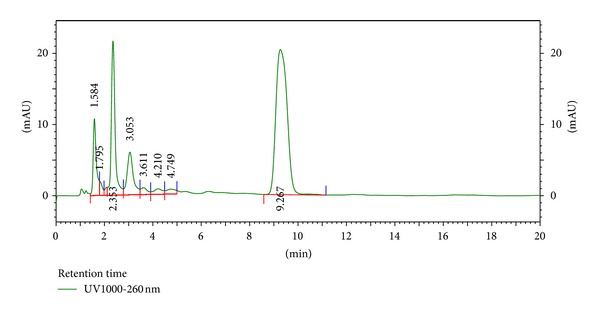
HPLC chromatogram (finger-printing) of methanol leaf extract (LWL) of *L. welwitschii *at *λ* = 254 nm.

The HPLC finger-printing of JFL and LWL extracts was determined to identify the major constituents in the various extracts for the identification and quality control purposes (Figures 1 and 2).

### 3.3. Antimicrobial Activity

The methanol leaf extracts (JFL and LWL) were found to be active against the test organisms with varying mean zones of inhibition. The MIC ranges of *J. flava *methanol extracts (JFL) against the test bacteria were 5.0 to 7.5 mg/mL and those of* L*. *welwitschii* extracts (LWL) were 2.5 to 10 mg/mL. The MBCs for the Gram-positive and Gram-negative bacteria were 10 to 20 mg/mL and 15 to 50 mg/mL, respectively (Table 2). Extracts (JFL and LWL) with concentrations of 20 and 50 mg/mL had higher zone of inhibition against the test organisms (Table 3). 

### 3.4. Antioxidant Activity

**Figure 3:**
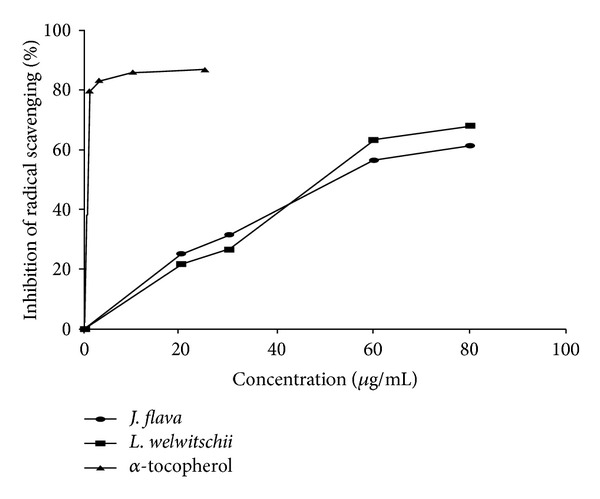
Free radical scavenging activities of methanol leaf extracts of *J. flava*, *L. welwitschii *and *α*-tocopherol (reference antioxidant) determined by DPPH method.

The extracts exhibited low antioxidant activities compared to the reference antioxidant, *α*-tocopherol (Table 4 and Figure 3).

### 3.5. Wound Healing Activity (Rate of Wound Closure)

**Figure 4:**
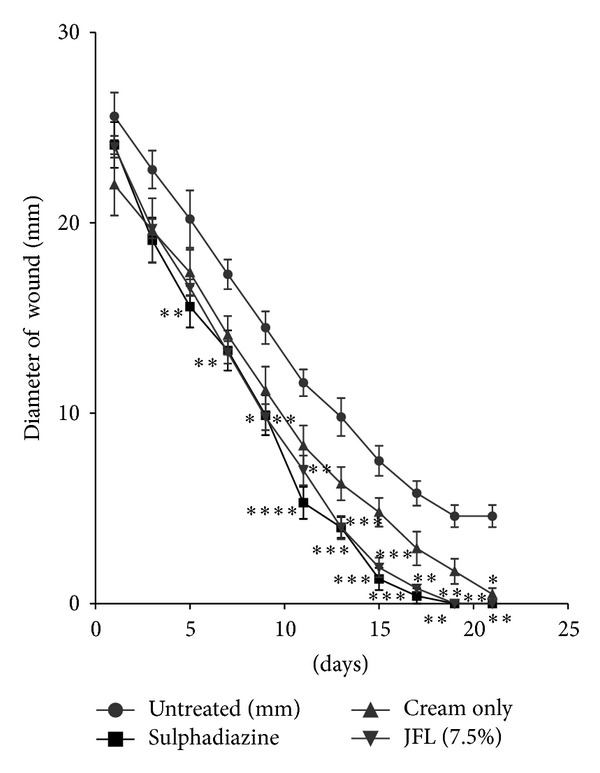
Influence of methanol leaf extract cream (7.5% w/w) of *J. flava *(JF) on the rate of closure of wounds. Values are diameter of wounds expressed as mean ± SEM (*N* = 5). *P < 0.05, **P < 0.01, and ***P < 0.001. Control is the untreated wounds. 1% w/w silver sulphadiazine was used as positive control.

**Figure 5:**
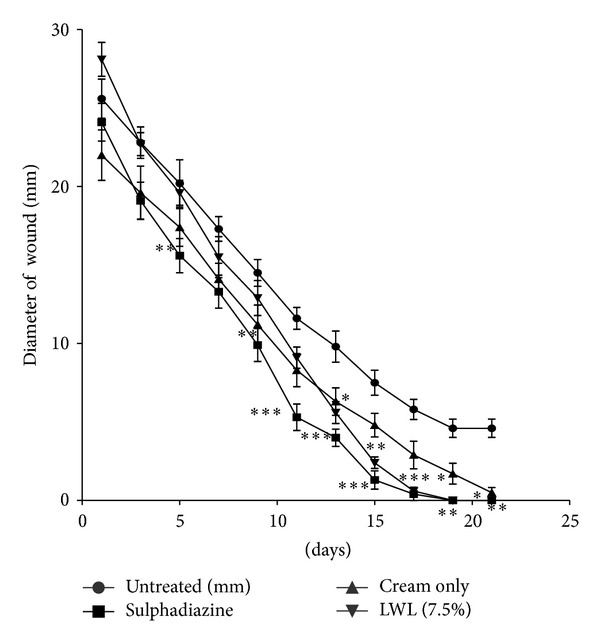
Influence of methanol leaf extract (LWL) cream (7.5% w/w) of *L. welwitschii *on the rate of closure of wounds. Values are diameter of wounds expressed as mean ± SEM (*N* = 5). *P < 0.05, **P < 0.01, and ***P < 0.001. Control is the untreated wounds. 1% w/w silver sulphadiazine was used as positive control.

Both extracts (JFL and LWL) increased the rate of wound closure compared with treated groups exhibited significant activities on the rates of wound closure compared to the untreated and the vehicle alone with JFL and LWL having significant influences (*P* < 0.05 and *P* < 0.01, resp.) on the rate of wound closure from 7th to 15th days after treatment (Figures 4 and 5, Table 5).

### 3.6. Tensile Strength of Wound Model

Tensile strengths for the treated group (7.5% w/w JFL and LWL extract creams) on the 10th day were found to be significant (*P* < 0.01) compared to the untreated group (Table 6).

### 3.7. Histopathological Examinations

**Figure 6:**
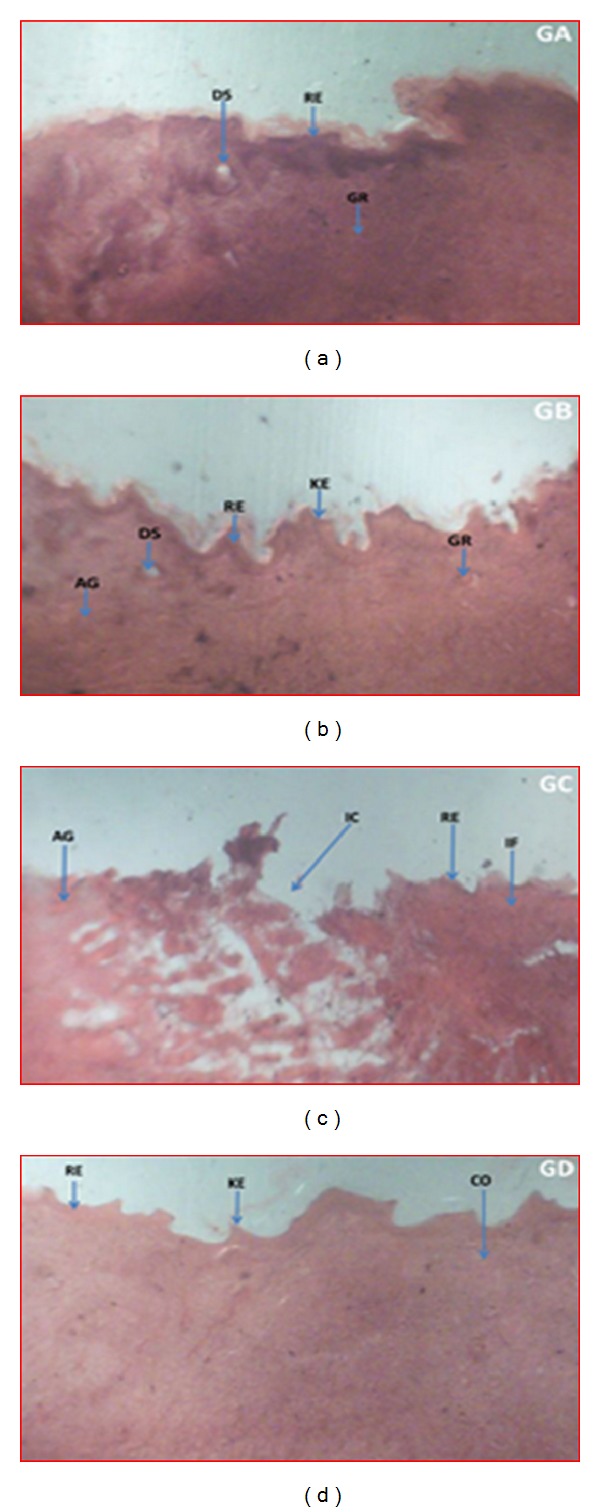
Histopathological examination of wound tissues treated with vehicle, extracts and the untreated tissues. Representative images stained with hematoxylin and eosin stain, Van Gieson's stain, and toluidine blue stain treated daily with 7.5% w/w creams of methanol leaf extracts of *J. flava* (JFL) (a) and *L. welwitschii *(LWL) (b) and the untreated wound tissues (c) for 14 days. (a) JFL: marked granulation tissue formation and angiogenesis with evidence of apoptosis due tissue necrosis. Complete wound resulted from increased collagenation and reepithelialisation. (b) LWL: enhanced collagenation and granulation tissue formation, evident of enhanced reepithelialisation and rate of healing, manifesting as uneven scaring. (c) Untreated wound tissues with persistent inflammation with incomplete wound area, evident of poor granulation tissue formation, collagenation, and reepithelialization but profuse angiogenesis. (d) Treated wound tissues (with 1% w/w silver sulphadiazine) having rapid granulation tissue formation and collagenation, evident of enhanced wound healing and uneven keratinous wound surface evident reepithelialization. AG: angiogenesis, CO: collagenation, DS: dead space following necrosis, GR: granulation tissue following apoptosis, IC: incomplete wound area, IF: inflamed tissue, KE: keratinous epithelium, ND: necrotic debris of persistent inflammation and RE: Reepithelialisation.

There was profuse proliferation of fibroblasts with varying degrees of fibrosis. The reference- and extract-treated wound tissues showed 60 to 75% dense with thickened fibrosis. Fibroblast cells and collagen fibers were more present in the reference and extracts treated groups as compared to untreated control. The creams containing 7.5% w/w of the extracts (JFL and LWL) showed enhanced angiogenesis, collagenation, and reepithelialization compared with the untreated wound tissues (Figure 6).

## 4. Discussion 

Several medicinal plants are used traditionally for the treatment of several microbial infections including boils, carbuncles, infected wounds, ulcers, diarrhea, dysentery, and so forth, and there is a need for ascertaining these claims scientifically. Methanol leaf extracts of *J. flava* and *L. welwitschii *were found to be active against the test organisms with *L. welwitschii *extract exhibiting more activity against compared to the *J. flava *extract. LWL extract had MIC range of 2.5 to 5 mg/mL against the Gram-positive bacteria and 5 to 10 mg/mL against the Gram-negative-bacteria. This result indicates the broad spectrum of activities of the extract, but the extract was less active against *P. aeruginosa.* JFL extract rather exhibited higher activity against the *P. aeruginosa* compared to LWL extract. The extracts (JFL and LWL) were found to be less active against the same test organisms when the agar well diffusion method was used to assess their antimicrobial activities. This may be attributed to the fact that the active principles or compounds from extracts were not able to diffuse into the agar medium and cause the necessary destruction of test organisms [20, 29]. The above findings may confirm the folkloric uses of the two plants as anti-infective agents.

Most wounds including ulcers are colonized by microorganisms, and this may lead to the infection of these wounds especially when they are inhabited with *S. aureus*, *Streptococcus *spp., and *P. aeruginosa *[30]. Infections stimulate an inflammatory response leading to persistent influx of neutrophils, and these polymorphonuclear neutrophils then release damaging substances such as free oxygen radicals and inflammatory mediators which tend to delay or prolong the wound healing process. Both extracts (JFL and LWL) exhibited good antimicrobial activity (Tables 2 and 3) and less antioxidant properties compared to the reference antioxidant, *α*-tocopherol (Table 4 and Figure 3). Antioxidants tend to speed up the healing process by destroying reactive oxygen species (ROS) [31]. Antioxidant properties of the extracts (JFL and LWL) might have contributed to the enhanced wound healing process. Plant extracts are used in treating wounds because of the effect of high levels of antioxidants [6].

Chronic infection of wounds reduces tensile strength especially in the proliferative phase. The methanol extracts were found to increase the tensile strength of the treated wounds significantly (*P* < 0.01) compared to the untreated wounds (Table 5). The increase in tensile strength of the treated wounds may be attributed to the increase in collagen biosynthesis and stabilization of the skin fibers [32].

There was significant difference between the influence of the extracts (JFL and LWL) on the rate wound closure compared to the untreated wounds. For *J. flava *cream (7.5% w/w), the rate of closure of the wounds was not significant within the first 8 days as compared to the untreated wounds. It showed more significant influence on the 9th day (*P* < 0.01). Percentages of wound closure on days 13 and 15 were much significant (*P* < 0.001) with wound closure of almost 100%. On days 15 and 17, few of the wounds were 100% closed with all the wounds closing on the 19th day. *L. welwitschii *cream (7.5% w/w) also showed wound healing activity but exhibited less activity compared to *J. flava *cream. There was not much closure by day 12 after the application of the cream. It rather showed more significant (*P* < 0.05) influence on the rate of contraction on the 13th day. There was much contraction in wounds in the subsequent days. Most of the wounds were fully closed on the 17th day with all the wounds closing on the 19th day. The late increase in wound contraction compared to the untreated group may be due to enhanced proliferation of fibroblasts and keratinocytes [25, 33] and subsequent marked increase in granulation tissue formation, angiogenesis, and reepithelialization of treated wounds compared to the untreated wounds (Figure 6).

The methanol leaf extracts were screened phytochemically for their secondary metabolites, and this revealed the presence of flavonoids, tannins, glycosides, and alkaloids. Some of these secondary metabolites such as flavonoids [34], alkaloids [35, 36], and tannins [33, 37, 38] have been found to influence wound healing in different *in vitro* and *in vivo* models. These metabolites, especially tannins, have been found also to possess antioxidant properties [39, 40]. Tannins have astringent and antimicrobial properties; hence the wound healing properties of *L. welwitschii *and *J. flava* may be attributed to it, and the increased wound contraction by the extracts could be a function of either the individual or the additive effects of the phytoconstituents [7, 25, 40]. There is a need to isolate and characterize the active principle(s) from the extracts which may be responsible for their antimicrobial and wound healing properties.

## 5. Conclusion

Methanol leaf extracts of *J. flava* and *L. welwitschii *were active against the test organism with MIC range of 2.5 to 10 mg/mL and MBC range of 10 to 50 mg/mL. The extracts significantly increased the tensile strength and the rate of contraction of wounds with intense granulation tissue formation, angiogenesis, and reepithelialization but exhibited less antioxidant activity. Phytochemical screening of both methanol leaf extracts of *J. flava *and *L. welwitschii *revealed the presence of tannins, flavonoids, alkaloids, and glycosides. The above findings may justify the traditional uses of these two plants for treatment of infections and as wound healing agents.

## Figures and Tables

**Table 1:** Phytochemical screening of dried leaves and methanol extracts of *J. flava* (JFL) and *L. welwitschii* (LWL).

Secondary metabolites	Alkaloids	Saponins	Flavonoids	Steroids	Carbohydrates	Sapogenetic glycosides	Tannins (% w/w)
Plant material/extract							
JFL leaf	+	−	+	+	+	+	0.94
LWL leaf	+	−	+	+	+	+	0.58

+: presence of secondary metabolite; −: absence of secondary metabolites.

**Table 2:** Minimum inhibitory concentrations (MIC) and minimum bactericidal/fungicidal concentrations (MBC/MFC) of methanol leaf extracts of *J. flava* (JFL) and *L. welwitschii* (LWL). The experiments were repeated three times.

Extract (mg/mL)	*S. aureus* ATCC 25923		*B. subtilis* NCTC 10073		*E. coli* ATCC 25922		*P. aeruginosa* ATCC 27853		*C. albicans *	
	MIC	MBC	MIC	MBC	MIC	MBC	MIC	MBC	MIC	MBC
JFL	5.0	10.0	7.5	20.0	7.5	20.0	7.5	20.0	5.0	10.0
LWL	5.0	10.0	2.5	7.5	5.0	15.0	10.0	50.0	2.5	5.0
CPC	0.025	nd	0.020	nd	0.025	nd	0.055	nd	nd	nd
CTZ	nd	nd	nd	nd	nd	nd	nd	nd	0.025	nd

Reference antimicrobial agents: CPC: chloramphenicol; CTZ: clotrimazole. nd: not determined.

**Table 3:** Antimicrobial activity of methanol leaf extracts of *J. flava* (JFL) and *L. welwitschii* (LWL) by agar diffusion method. Mean zones of growth inhibition (plus diameter of well) are mean (mm) of 3 independent experiments, mean ± SD, *n* = 3 replicates, and diameter of well/cup = 8 mm.

Mean zones of growth inhibition (mm)					
Extract (mg/mL)	Test organisms				
	*S. aureus* ATCC 25923	*B. subtilis* NCTC 10073	*E. coli* ATCC 25922	*P. aeruginosa* ATCC 27853	*C. albicans *
JFL					
10	10.00 ± 0.50	10.50 ± 0.50	10.00 ± 0.50	10.00 ± 0.50	11.50 ± 0.50
20	10.50 ± 0.50	12.00 ± 0.50	11.50 ± 0.45	12.00 ± 0.50	12.50 ± 0.55
50	12.50 ± 0.45	14.50 ± 0.45	13.55 ± 0.50	13.50 ± 0.50	15.50 ± 0.50
LWL					
10	10.50 ± 0.50	13.50 ± 0.50	14.50 ± 0.50	0.0	11.50 ± 0.50
20	12.00 ± 0.50	15.50 ± 0.55	19.50 ± 0.50	12.00 ± 0.50	13.50 ± 0.50
50	15.50 ± 0.50	18.00 ± 0.50	25.55 ± 0.50	14.50 ± 0.55	15.50 ± 0.50
CPC	25.5 ± 0.50	31.00 ± 0.50	30.50 ± 0.50	17.50 ± 0.50	nd
CTZ	nd	nd	nd	nd	25.50 ± 0.50

Reference antimicrobial agents: CPC: chloramphenicol (1 mg/mL); CTZ: clotrimazole (1 mg/mL). nd: not determined.

**Table 4:** Free radical scavenging activities of methanol leaf extracts of *J. flava* (JFL) and *L. welwitschii* (LWL) and *α*-tocopherol determined by DPPH method.

Extracts	IC_50_ (*µ*g/mL)
JFL	65.3
LWL	81.8
*α*-tocopherol	1.5

**Table 5:** Summary of wound closures for selected time points. Values aremean wound area (mm^2^) ± SEM for untreated wounds and wounds treated with 1% w/w silver sulphadiazine, 7.5% w/w *J. flava* extract cream (JF), 7.5% w/w *L. welwitschii* extract cream (LWL), and cream only. *N* = 5 rats per group. Data analysed by one-way ANOVA followed by Dunnett's multiple comparison test.

Day	Untreated wounds	Silver sulphadiazine (1% w/w)	Cream only	LWL (7.5% w/w)	JF (7.5% w/w)
1	519.62 ± 113.01	460.67 ± 100.78	388.22 ± 119.65	623.80 ± 101.56	453.41 ± 48.99
3	400.59 ± 72.97	290.87 ± 77.59	310.71 ± 111.47	406.40 ± 58.77	374.91 ± 163.66
5	375.50 ± 117.88	194.86 ± 58.56**	242.29 ± 74.09	300.69 ± 55.28	219.87 ± 30.85
7	236.99 ± 45.97	142.43 ± 52.16	159.32 ± 51.24	194.11 ± 71.80	137.92 ± 27.16
9	167.41 ± 42.92	80.47 ± 39.52**	103.44 ± 48.03	134.58 ± 49.26	76.89 ± 23.31**
11	112.74 ± 28.62	24.31 ± 18.70***	57.61 ± 29.41	66.48 ± 21.49*	40.37 ± 18.00**
13	73.00 ± 26.54	13.51 ± 8.70***	33.58 ± 18.89	26.15 ± 12.00**	13.74 ± 8.15***
15	46.14 ± 20.08	2.40 ± 2.93***	19.87 ± 12.15	4.95 ± 3.28**	3.65 ± 2.60***
17	27.72 ± 13.20	0.63 ± 1.40**	9.07 ± 8.29	0.32 ± 0.43**	0.63 ± 0.35**
19	17.67 ± 9.75	0	3.65 ± 4.39**	0	0
21	17.67 ± 9.57	0	0.51 ± 0.78**	0	0

*P < 0.05, **P < 0.01, and ***P < 0.001 were considered statistically significant compared to the untreated wounds.

**Table 6:** Influence of methanol leaf extracts of *J. flava* (JFL) and *L. welwitschii* (LWL) on tensile strength of wound tissues. Values represent mean ± SEM; *N* = 5 rats per group.

Wound type	Tensile strength (g/mm^2^)
Untreated wounds	432.50 ± 5.63
Wounds treated with 1% w/w silver sulphadiazine	616.46 ± 4.21*
Cream alone	439.85 ± 3.24
Wounds treated with JFL (7.5% w/w) cream	578.47 ± 4.64*
Wounds treated with LWL (7.5% w/w) cream	590.32 ± 5.64*

*P < 0.01.
